# Pediatric Psoriasis with or without Arthritis: Does It Make a Difference?

**DOI:** 10.3390/jcm13010242

**Published:** 2023-12-31

**Authors:** Ayelet Ollech, Mor Rotenberg, Irit Tirosh, Efrat Bar-Ilan, Michal Solomon, Shoshana Greenberger, Felix Pavlotsky

**Affiliations:** 1 Pediatric Dermatology Unit, Dermatology Department, Sheba Medical Center, Ramat Gan 5262160, Israelshoshana.greenberger@sheba.health.gov.il (S.G.); 2 Faculty of Medicine, Tel Aviv University, Tel Aviv 6997801, Israelfelixp@tauex.tau.ac.il (F.P.); 3 Department of Dermatology, Hadassah Medical Center, The Faculty of Medicine, Hebrew University of Jerusalem, Jerusalem 9112102, Israel; 4 Pediatric Rheumatology Unit, Edmond and Liliy Safra Medical Center, Jerusalem 9112102, Israel; 5 Department of Dermatology, Sheba Medical Center, Tel Hashomer 5262160, Israel; 6 Psoriasis and Phototherapy Center, Sheba Medical Center, Tel Hashomer 5262160, Israel

**Keywords:** psoriasis, psoriatic arthritis, pediatrics, joints, juvenile inflammatory arthritis, systemic

## Abstract

Background: Psoriasis and psoriatic arthritis can present simultaneously or separately in children and may pose a diagnostic challenge. Objective: To compare the dermatological manifestations in pediatric psoriatic patients with and without arthritis. Methods: A retrospective case-control study of psoriatic patients ≤ 18 years old at Sheba Medical Center was conducted between 2011 and 2021. Patients with psoriatic arthritis versus psoriasis-only were compared according to body surface area (BSA) involvement, cutaneous distribution, severity of skin disease, response to treatment and related side effects. Results: The study cohort included 29 psoriatic arthritis and 64 psoriasis-only patients matched by age and sex. The psoriasis-only group had a significantly higher mean BSA (19.7%, SD ± 18.7) than the psoriatic arthritis group (6.1%, SD ± 11.4), (*p* = 0.029). The skin distribution differed with the psoriasis group showing more involvement of the extremities, scalp, trunk, and genitals. Both groups primarily experienced partial responses to methotrexate, whereas the psoriasis group mainly saw complete responses to biologics. Adverse events were rare, with a higher incidence in the psoriasis group. Conclusion: This retrospective study highlights the differences in cutaneous disease characteristics, severity, and treatment response in pediatric patients with psoriasis and psoriatic arthritis, providing valuable insights for diagnosis and disease course in the pediatric population.

## 1. Introduction

Psoriasis is a multifactorial disorder characterized by erythematous, scaly skin lesions, while psoriatic arthritis encompasses joint and/or other musculoskeletal inflammation with or without skin lesions [1]. The onset of both conditions during childhood can have significant implications for the affected child’s quality of life, social functioning, and long-term health outcomes [2,3,4].

The prevalence of psoriasis in children ≤ 18 years of age varies across different geographic regions, but estimates suggest that the prevalence of psoriasis in the pediatric population is approximately 1% [5,6]. Psoriasis in children often exhibits unique clinical characteristics; lesions are more commonly localized to the face, scalp and/or are present in guttate or pustular forms [1].

Juvenile psoriatic arthritis (JPsA) affects 6–8% of pediatric patients with inflammatory arthritis and 0.7–1.2% of pediatric psoriasis patients [7]. The age of onset is bimodal, with peaks in early childhood (2–3 years) and during adolescence (10–12 years) [8]. Younger children, especially girls, are more likely to exhibit symptoms of oligoarticular disease and/or dactylitis. On the other hand, older children, particularly boys, are more prone to exhibit enthesitis and axial disease. Dactylitis, nail changes, and uveitis are additional features observed in JPsA [9,10,11].

Diagnosing psoriasis and JPsA in children can be particularly challenging due to possible atypical presentation, potential overlap with other dermatological and rheumatological conditions, and the limited number of diagnostic criteria tailored for the pediatric population. 

Understanding the unique clinical features, diagnostic challenges, and therapeutic considerations of psoriasis and psoriatic arthritis in children is crucial for timely diagnosis and optimal management. Specifically, it is unclear whether psoriatic skin lesions present differently in patients with and without arthritis and whether these lesions respond to therapy similarly.

This study aimed to compare the dermatological manifestations and their severity in pediatric psoriatic patients with and without arthritis.

## 2. Methods

A retrospective case-control study, conducted at the dermatology and pediatric rheumatology clinics in the Sheba Medical Center, Tel Hashomer between the years 2011 and 2021. Patients < 18 years of age diagnosed with psoriasis and/or psoriatic arthritis were included.

Data of patients diagnosed with psoriatic arthritis (PSA group) compared to a sex and age-matched pool of patients with the diagnosis of psoriasis without arthritis (PSO group) were included in the analysis.

Data were collected from the medical files of the patients and included sex, age, age at diagnosis of psoriasis and/or psoriatic arthritis, past medical and treatment history, familial history of psoriasis or arthritis, psoriasis main clinical subtype (plaque, guttate, pustular, or inversa), body surface area (BSA) and special location involvement (scalp, genitals, palmoplantar, nails), subtype of joint involvement (oligoarthritis, polyarthritis, sacroiliitis) and location, disease severity (detailed below), treatment, disease course, outcome (detailed below) and adverse events. 

Disease severity was defined by the investigator global assessment (IGA) scale (scale 0–3), response to treatment was defined as complete response (CR), partial response (PR) and no response (NR) in accordance with the change in IGA scores (CR, >2 points change, PR, 1–2 points change, NR, no change or worsening). 

### Statistical Analysis

The data were first inspected descriptively to understand the characteristics of the cohort. The age distribution for PSA for each sex, was analyzed with the Gaussian mixture model (GMM) fits overlaid.

The median affected BSA was compared between the PSA and PSO groups, including only the cases who presented with a cutaneous rash in the PSA group, using the non-parametric Mann–Whitney U test due to the non-normal distribution of the data.

The involved skin area was compared between the PSA and PSO groups using the chi-square test of independence. The same was used to compare the severity between the two groups and the association between gender and the involved skin area.

Disease severity was examined using ordinal logistic regression models according to family history of psoriasis, age, and gender. 

Lastly, each treatment response was stratified by disease group (PSA or PSO).

All statistical analyses were performed using SPSS software (IBM SPSS Statistics for Windows Version 25, IBM Corporation, Armonk, NY, USA 2017). A *p*-value of less than 0.05 was considered statistically significant.

The study was approved by the local ethics committee (IRB-6178-19SMC) approval date 3 September 2019.

## 3. Results

### 3.1. Patient Characteristics

#### 3.1.1. Psoriatic Arthritis Group (PSA)

**Figure 1:**
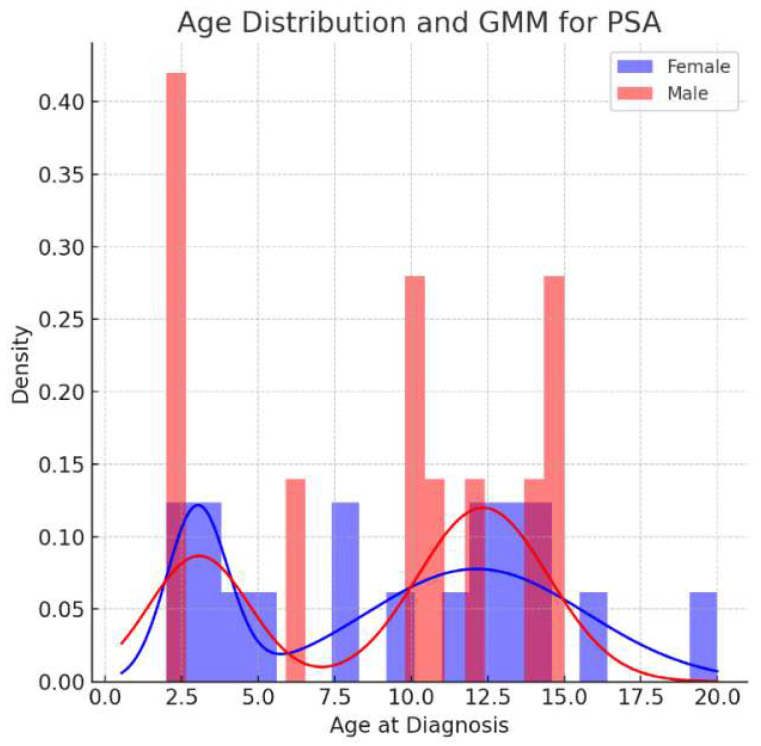
The age distribution for psoriatic arthritis for each sex.

There were twenty-nine pediatric patients diagnosed with psoriatic arthritis, 18 (62%) females, with a mean age of 15.3 (±4.6) years and a mean follow up of 2.85 (±2.9) months. The age distribution for each gender showed a bimodal distribution (Figure 1). The dermatological disease was diagnosed at a mean age of 9.2 (±5.1, range 2–17) years, and the rheumatological diagnosis made at the mean age of 10.1 (±5.2, range 2–16) years. 

##### GMM, Gaussian Mixture Model, PSA—Psoriatic Arthritis

In 15 (51%) cases, the rheumatological disease was diagnosed first, and after a mean of 4.9 (±3.9) years, the psoriatic skin involvement occurred, whereas in 14 (48%) cases, the dermatological disease was diagnosed first and after a mean of 3.8 (±3.5) years, the joint involvement followed. 

In this group, the diagnosis of psoriatic arthritis was made by a pediatric rheumatologist in all cases, while the diagnosis of the psoriasis was made by a dermatologist in only 16 (55%) cases and the remaining cases were diagnosed by a rheumatologist or a general practitioner in 12 (41%) and 1 (3%) case, respectively. In 6 (21%) cases, the diagnosis of psoriatic arthritis was made without psoriatic skin rash, based on the presence of dactylitis and/or nail pitting. 

#### 3.1.2. Psoriasis-Only Group (PSO)

A total of 64 age- and sex-matched patients with psoriasis without arthritis, females 45 (70%) ratio, a mean age at data collection of 14.1 (±6.3) years, mean age at diagnosis of 10.1 (0.9–18) years and a follow up period of 3.3 (0.04–4) years.

Their past medical and familial background, psoriasis and joint involvement and severity of diseases are presented in Table 1.

### 3.2. Comparison of PSA and PSO Groups

#### 3.2.1. Body Surface Area Affected by Psoriasis

**Figure 2:**
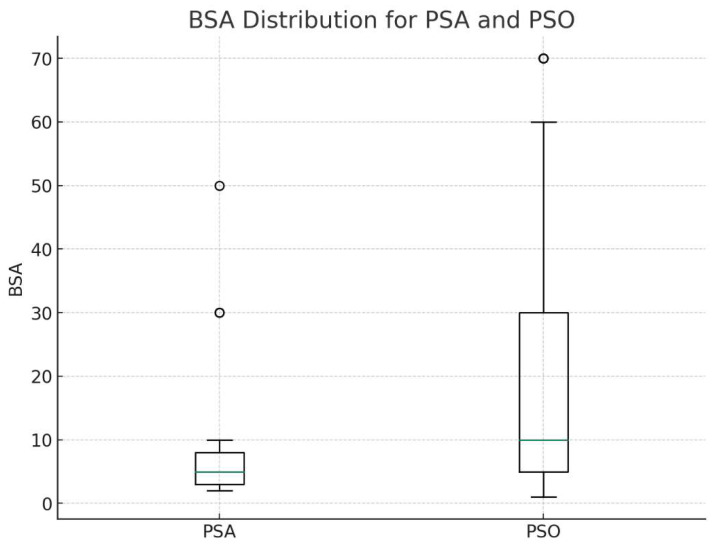
The distribution of body surface area in the psoriasis and psoriatic arthritis groups. BSA—body surface area, PSO—psoriasis group, PSA—psoriatic arthritis group. Whisker plot showing outliers, 5–95% CI, The interquartile range and median values.

The median BSA affected by psoriasis was significantly higher in the PS0 group (19.7%) compared to the PSA group (6.1%) (*p* = 0.029) (Figure 2). To note, in six cases (20%) of the PSA group, there was no psoriatic skin rash; in 2 (6.8%) cases, the only finding was nail pitting.

#### 3.2.2. Anatomical Distribution of Psoriasis

**Figure 3:**
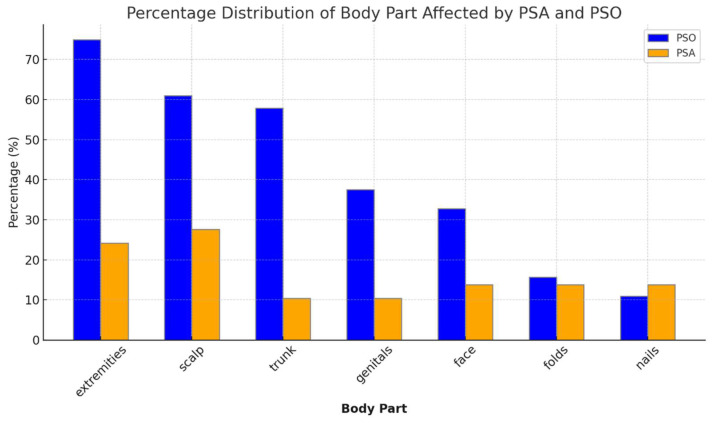
The distribution of psoriasis by body area in the psoriasis and psoriatic arthritis groups.

Anatomical distribution included extremities (75% vs. 24%), the scalp (61% vs. 28%), trunk (58% vs. 10%), genitals (38% vs. 10%), face (33% vs. 14%), skin folds (16% vs. 14%) and nail involvement (11% vs. 14%) in PSO and PSA groups, respectively. Statistically significant differences between the groups (*p* < 0.05) were observed in the distribution of the disease in the scalp, trunk, extremities, and genitals. In the PSA group, the scalp followed by the extremities were the most common areas with psoriasis (Figure 3).

For both genders in the entire cohort, the extremities were most commonly involved. Females had a higher rate of the scalp 59% (*p* = 0.0386) and facial 35% (*p* = 0.0224) involvement compared to 33% and 10% in males, respectively.

#### 3.2.3. Severity and Response to Treatment

The severity of both diseases is presented in Table 1. Most patients had mild to moderate skin disease (77% in the PSO group and 70% in the PSA group). To note, nine cases in the PSA group were not included (in six, there was no skin involvement, and in three, there was missing data). The difference in severity distributions between the two groups was not statistically significant. There was no evidence to suggest that family history of psoriasis, age at diagnosis, or sex were significantly related to psoriasis severity.

Most patients received topical treatments as a first line treatment with variable responses, mostly PR (66% in the PSO vs. 71% in the PSA group) with only a few cases achieving CR, mainly mild disease. Phototherapy therapy was received by 61% patients in the PSO group and 17% of the PSA group and achieved mainly PR (52.5% in the PSO vs. 60% in the PSA group) followed by CR (30.6% in the PSO vs. 40% in the PSA group).

Responses for each group (PSO vs. PSA) to systemic therapies (MTX or biologics) indicated for the skin disease, showed PR in the PSA group (60% or 75%, respectively) followed by NR (20% or 25%, respectively). In the PSO group, the majority of patients achieved CR to biologics (60%) and PR to MTX (50%). The responses to the systemic treatments were not statistically significant. To note, a PSA patient who received tofacitinib treatment for the arthritis had only a PR for the psoriasis.

The response to each treatment stratified in the PSA and PSO groups is presented in Table 2.

In the PSA group with no record of skin involvement, five of the patients were on systemic treatments for the arthritis including: MTX (one patient), biological therapy (three patients) or combination (two patients). One patient had only nail pitting without other skin findings nor any systemic treatment for his psoriatic arthritis. 

#### 3.2.4. Adverse Events

Adverse events (AEs) from the systemic therapies were rare and not severe; however, more common in the psoriasis group compared to the psoriatic arthritis group.

In the PSA group, there were gastrointestinal AEs under MTX in six cases, Herpes Zoster infection under tofacitinib (1 case) and a pustular psoriasis eruption following adalimumab (1 case). In the PSO group, there were two cases of gastrointestinal AEs and two case of elevated liver transaminases (grades 1 and 3), one case of Bell’s palsy under etanercept and two cases of paradoxical psoriasiform eruption under adalimumab. There was xerosis with skin fissuring under one case treated with acitretin.

The medication was stopped in two cases, in the case of Bell’s palsy following one dose of etanercept (PSO group) and in the case of gastrointestinal AE following MTX (PSA group).

## 4. Discussion

We present a 10-year cohort of ninety-four pediatric patients diagnosed with either psoriasis and/or psoriatic arthritis. Notably, the age of onset for psoriasis and psoriatic arthritis was found to be early or bimodal, with peaks in early childhood and adolescence, underscoring the importance of early detection and management.

In our study group, the joint disease preceded or was simultaneously diagnosed with the skin diseases in 52%, which is lower than described in previous studies [1,12].

The study identified notable differences in clinical characteristics between pediatric patients diagnosed with psoriasis alone and those with concurrent psoriatic arthritis. Noteworthy was the significant difference in BSA affected by psoriasis between these two groups in favor of the psoriasis group. However, in 20% of the psoriatic arthritis group, there was no cutaneous skin disease involvement, with only findings of nail pitting (13%).

In adult cases, including most studies in meta-analysis [13,14], patients with severe skin disease are up to two times more likely to have joint disease. In this population, psoriasis typically precedes or is diagnosed concurrently with arthritis in 85% of cases [11]. Patients diagnosed with psoriasis in childhood may develop arthritis years later, when they are adults.

However, in children, the pattern is reversed: 80% of children with psoriatic arthritis experience joint inflammation prior to skin disease, typically 2–3 years earlier and up to a decade behind arthritis onset, complicating diagnosis [7].

An analysis of 361 children from the CARRA-JPsA registry showed that children with JPsA have a lower incidence of psoriasis compared to adults [9]. Similar findings have been reported in other JPsA studies, which aligns with our study [10,15]. It is speculated that this could be because, unlike in adult psoriatic-arthritis patients, children with JPsA usually develop psoriasis several years after the onset of arthritis, in the absence of typical psoriatic lesions. The International League of Associations of Rheumatology (ILAR) criteria for the diagnosis of JPsA includes at least two of the following: dactylitis, nail pitting, onycholysis or family history of psoriasis in a first-degree relative [11,16] and, therefore, psoriatic arthritis can be diagnosed without a skin rash. Additionally, the emergence of psoriasis may be masked by the use of systemic therapies [11].

Another key finding was the variability in the body area distribution of psoriasis. Based on our study, as a common finding (28%) amongst the psoriatic arthritis group, pediatric patients with the suspicion or diagnosis of psoriatic arthritis should be examined for scalp lesions. In all cases of suspicion of either disease, nail examination is mandatory. These differences warrant further investigation to determine their predictive value and to enhance diagnostic accuracy.

In pediatric psoriasis, scalp involvement is described in 79% of the patients, more commonly in girls, similar to our findings. Nail changes are described in 39% of pediatric psoriasis cases and are more common in boys [5]. In JPsA, nail involvement is reported in 57% of the cases. The prevalence of dactylitis (29.7%) was lower compared to previous reports. The prevalence of enthesitis and uveitis are in the range of 14–45% of published series [9,10,17].

The therapeutic interventions for psoriasis may include topical agents, phototherapy, systemic immunomodulatory medications, and in severe cases, biologic agents should be considered as an advanced-line treatment [1,18].

Biological treatments for pediatric psoriasis approved by the FDA and EMA include anti-TNF agents such as etanercept and adalimumab as well as the anti-IL-12/23 ustekinumab. The anti-IL-17 secukinumab and anti-IL-23 ixekizumab, while extensively evidenced in the adult population are gradually gaining evidence for their effectiveness in the pediatric population [19]. These agents, with their specific targeting, high efficacy, and favorable safety profiles, offer favorable therapeutic options [19].

For JPsA, non-steroidal anti-inflammatory drugs and oral glucocorticoids, as well as intra-articular glucocorticoids, are indicated as initial steps, whereas disease-modifying antirheumatic drugs (DMARDs) represent the mainstay treatment. The most used is MTX, which is usually well tolerated in children. In severe cases, biologic agents should be considered as an advanced line therapy. Recently, the Janus kinase inhibitor tofacitinib, has been approved for JIA and JPsA [7].

In the case of psoriasis and psoriatic arthritis, the therapy is preferably aimed for both, with a benefit/risk balance to affect both aspects of the disease—skin and joints—in accordance with their severity.

In our study, most patients received topical treatments as first line, with variable results. The use of systemic treatments, such as MTX appeared to elicit a PR in most patients in both the psoriasis and the psoriatic arthritis group, whereas biological agents, indicated for the skin in the psoriasis group only, resulted mostly in CR.

This suggests that systemic treatments could have enhanced effectiveness in managing psoriasis, although patient-tailored approaches are paramount due to varying individual responses.

Interestingly, the majority of patients in the psoriatic arthritis group who had no skin involvement were under systemic therapy for their arthritis, suggesting that the systemic treatment may have had a “protective” effect on the skin disease. On the other hand, the MTX and/or biologic agent doses used for the psoriatic arthritis group are lower in many cases than those needed for psoriasis.

## 5. Limitations

However, this study is not without limitations. Due to the limited number of cases, it was challenging to establish a correlation between early intervention with a systemic agent for either psoriasis or psoriatic arthritis and its impact on delaying or influencing the severity of the other condition’s presentation.

Additional limitations include a small sample size, its retrospective nature, and missing data, which can lead to selection and information biases. The diagnosis of psoriasis made by non-dermatologists in some cases, could potentially affect the precision of these diagnoses. Further prospective studies with larger sample sizes and comprehensive data collection are needed to validate these findings.

## 6. Conclusions

This study represents a significant advancement in our understanding of pediatric psoriasis and psoriatic arthritis, highlighting the critical importance of early and accurate diagnosis and the need for tailored therapeutic approaches. Future research is warranted to delve deeper into the pathogenesis of these diseases, to develop reliable diagnostic tools, and potentially influence the clinical guidelines to enhance the management, outcomes, and quality of life for affected children.

## Figures and Tables

**Table 1:** Past medical and familial background, psoriasis and joint involvement and disease severity.

	Psoriatic Arthritis (n, %)	Psoriasis Only (n, %)
Total	29	64
Past medical history	Depression 2 (6.8) Hypermobility 2 (6.8) Other 1 each (3.4) (ADHD, HS, celiac, CRPS, precocious puberty) Uveitis 4 (13.7)	Atopy (asthma, AD) 4 (6.3) ADHD 3 (4.7) Down syndrome (4.7) Eating disorder 2 (3.1) Short stature 2 (3.1) Other 1 each (1.5) (migraine, celiac, Crohn’s, hypothyroidism, metabolic syndrome, seizures)
Labs	Positive ANA 7 (24.1)	Positive strep A test 5 (7.8)
Family history psoriasis	20 (68) 1st 10 (34.4) 2nd 6 (20.6) 1st + 2nd 4 (13.7)	48 (75) 1st 37 (57.8) 2nd 11 (17.2)
Family history arthritis	6 (20%) RA 3 (10%)	NA
Psoriasis subtype	Plaque 23 (79) No skin lesions 6 (20.6)	Plaque 51 (79.7) Guttate 5 (7.8) Inversa 3 (4.7) Nail 2 (3.1) Palmoplantar 2 (3.1)
Psoriasis involvement	Scalp 13 (44.8) Face, ears 4 (13.8) Trunk 5 (17.2) Extremities 12 (41.3) Inversa 6 (20.7) Nails 6 (20.7) Dactylitis 10 (34.4)	Scalp 39 (60.9) Face, ears 21 (32.8) Trunk 37 (57.8) Extremities 28 (75) Inversa 24 (37.5) Nails 8 (12.5)
Skin involvement severity	Severe 6 (30%) Mild–moderate 14 (70%)	Severe 14 (22%) Mild–moderate 46 (76%)
Psoriatic arthritis subtype	Oligoarthritic 19 (65.5) Polyarthritis 8 (27.5) Sacroiliitis 2 (0.07)	
Joint involvement	Elbow-4 (6.5) Hands-7 (11.4) Wrists-8 (13.2) Feet-5 (8.1) Ankle 13 (21.3)	
Joint disease severity	Mild–moderate 7 (24%) Severe 22 (76%)	

ADHD—attention deficit disorder, HS—hidradenitis suppurativa, CRPS—complex regional pain syndrome, AD—atopic dermatitis, ANA—antinuclear antibody, RA—rheumatoid arthritis, NA—not available.

**Table 2:** The response to each skin directed treatment in the psoriasis and psoriatic arthritis groups.

Treatment	Disease	No Response (n, %)	Complete Response (n, %)	Partial Response (n, %)	Missing Data (n, % of Total)	Total Patients
MTX	PSO	1 (25%)	1 (25%)	2 (50%)	3 (43%)	7 (11%)
	PSA	1 (20%)	1 (20%)	3 (60%)	-	5 (17%
Biological	PSO	-	3 (60%)	2 (40%)	-	5 (8%)
	PSA	1 (25%)	-	3 (75%)	-	4 (13.8%)
Phototherapy	PSO	1 (3%)	12 (35%)	21 (62%)	6 (15%)	40 (61.5%)
	PSA	-	2 (40%)	3 (60%)	-	5 (17%)
Acitretin	PSO	1 (25%)	2 (50%)	1 (25%)	4 (50%)	8 (12.5%)

PSO—psoriasis, PSA—psoriatic arthritis, MTX—methotrexate.

## Data Availability

Data is contained within the article.

## References

[1] Menter A., Cordoro K.M., Davis D.M.R., Kroshinsky D., Paller A.S., Armstrong A.W., Connor C., Elewski B.E., Gelfand J.M., Gordon K.B. (2020). Joint American Academy of Dermatology-National Psoriasis Foundation guidelines of care for the management and treatment of psoriasis in pediatric patients. J. Am. Acad. Dermatol..

[2] Imhof R.L., Eton D.T., Tollefson M.M. (2023). The impact of childhood psoriasis on the quality of life of parents and caregivers. Pediatr. Dermatol..

[3] Mahe E., Maccari F., Beauchet A., Lahfa M., Barthelemy H., Reguiai Z., Beneton N., Estève E., Chaby G., Ruer-Mulard M. (2013). Childhood-onset psoriasis: Association with future cardiovascular and metabolic comorbidities. Br. J. Dermatol..

[4] Rustad A.M., Nolan B.E., Ollech A., Boctor M.J., Paller A.S. (2021). Incorporating joint pain screening into the pediatric dermatologic examination. Pediatr. Dermatol..

[5] Mercy K., Kwasny M., Cordoro K.M., Menter A., Tom W.L., Korman N., Belazarian L., Armstrong A.W., Levy M.L., Paller A.S. (2013). Clinical manifestations of pediatric psoriasis: Results of a multicenter study in the United States. Pediatr. Dermatol..

[6] Tollefson M.M., Crowson C.S., McEvoy M.T., Maradit Kremers H. (2010). Incidence of psoriasis in children: A population-based study. J. Am. Acad. Dermatol..

[7] Brunello F., Tirelli F., Pegoraro L., Dell’Apa F., Alfisi A., Calzamatta G., Folisi C., Zulian F. (2022). New Insights on Juvenile Psoriatic Arthritis. Front. Pediatr..

[8] Petty R.E., Southwood T.R., Manners P., Baum J., Glass D.N., Goldenberg J., He X., Maldonado-Cocco J., Orozco-Alcala J., Prieur A.-M. (2004). International League of Associations for Rheumatology classification of juvenile idiopathic arthritis: Second revision, Edmonton, 2001. J. Rheumatol..

[9] Shore A., Ansell B.M. (1982). Juvenile psoriatic arthritis--an analysis of 60 cases. J. Pediatr..

[10] Stoll M.L., Zurakowski D., Nigrovic L.E., Nichols D.P., Sundel R.P., Nigrovic P.A. (2006). Patients with juvenile psoriatic arthritis comprise two distinct populations. Arthritis Rheum..

[11] Zisman D., Gladman D.D., Stoll M.L., Strand V., Lavi I., Hsu J.J., Mellins E.D., CARRA Legacy Registry Investigators (2017). The Juvenile Psoriatic Arthritis Cohort in the CARRA Registry: Clinical Characteristics, Classification, and Outcomes. J. Rheumatol..

[12] Brandon T.G., Manos C.K., Xiao R., Ogdie A., Weiss P.F. (2018). Pediatric psoriatic arthritis: A population-based cohort study of risk factors for onset and subsequent risk of inflammatory comorbidities. J. Psoriasis Psoriatic Arthritis.

[13] Mease P.J., Etzel C.J., Huster W.J., Muram T.M., Armstrong A.W., Lisse J.R., Rebello S., Dodge R., Murage M.J., Greenberg J.D. (2019). Understanding the association between skin involvement and joint activity in patients with psoriatic arthritis: Experience from the Corrona Registry. RMD Open.

[14] Pouw J.N., Jacobs M.E., Balak D.M.W., van Laar J.M., Welsing P.M.J., Leijten E.F.A. (2022). Do Patients with Psoriatic Arthritis Have More Severe Skin Disease than Patients with Psoriasis Only? A Systematic Review and Meta-Analysis. Dermatology.

[15] Southwood T.R., Petty R.E., Malleson P.N., Delgado E.A., Hunt D.W., Wood B., Schroeder M.-L. (1989). Psoriatic arthritis in children. Arthritis Rheum..

[16] Zabotti A., De Marco G., Gossec L., Baraliakos X., Aletaha D., Iagnocco A., Gisondi P., Balint P.V., Bertheussen H., Boehncke W.-H. (2023). EULAR points to consider for the definition of clinical and imaging features suspicious for progression from psoriasis to psoriatic arthritis. Ann. Rheum. Dis..

[17] Butbul Aviel Y., Tyrrell P., Schneider R., Dhillon S., Feldman B.M., Laxer R., Saurenmann R.K., Spiegel L., Cameron B., Tse S.M.L. (2013). Juvenile Psoriatic Arthritis (JPsA): Juvenile arthritis with psoriasis?. Pediatr. Rheumatol. Online J..

[18] Bronckers I., Paller A.S., West D.P., Lara-Corrales I., Tollefson M.M., Tom W.L., Hogeling M., Belazarian L., Zachariae C., Mahé E. (2020). A Comparison of Psoriasis Severity in Pediatric Patients Treated With Methotrexate vs. Biologic Agents. JAMA Dermatol..

[19] Megna M., Camela E., Battista T., Genco L., Martora F., Noto M., Picone P., Ruggiero A., Monfrecola G., Fabbrocini G. (2023). Efficacy and safety of biologics and small molecules for psoriasis in pediatric and geriatric populations. Part I: Focus on pediatric patients. Expert Opin. Drug Saf..

